# Long range electronic phase separation in CaFe_3_O_5_

**DOI:** 10.1038/s41467-018-05363-6

**Published:** 2018-07-30

**Authors:** Ka. H. Hong, Angel M. Arevalo-Lopez, James Cumby, Clemens Ritter, J. Paul Attfield

**Affiliations:** 1 Centre for Science at Extreme Conditions and School of Chemistry, University of Edinburgh, Mayfield Road, Edinburgh, EH9 3JZ UK; 2 Univ. Lille, CNRS, Centrale Lille, ENSCL, Univ. Artois, UMR 8181 - UCCS - Unité de Catalyse et Chimie du Solide, Lille, F-59000 France; 3 Institut Laue-Langevin, 71 avenue des Martyrs, Grenoble, 38000 France

## Abstract

Incomplete transformations from ferromagnetic to charge ordered states in manganite perovskites lead to phase-separated microstructures showing colossal magnetoresistances. However, it is unclear whether electronic matter can show spontaneous separation into multiple phases distinct from the high temperature state. Here we show that paramagnetic CaFe_3_O_5_ undergoes separation into two phases with different electronic and spin orders below their joint magnetic transition at 302 K. One phase is charge, orbital and trimeron ordered similar to the ground state of magnetite, Fe_3_O_4_, while the other has Fe^2+^/Fe^3+^charge averaging. Lattice symmetry is unchanged but differing strains from the electronic orders probably drive the phase separation. Complex low symmetry materials like CaFe_3_O_5_ where charge can be redistributed between distinct cation sites offer possibilities for the generation and control of electronic phase separated nanostructures.

## Abstract (web summary)

Electronic phase separation is an important feature of many correlated perovskite compounds but hasn’t been seen in other complex oxides with similar physical behaviour such as magnetite. Hong et al. find phase separation between a magnetite-like charge ordered phase and a charge averaged phase in CaFe_3_O_5_.

## Introduction

Manganites such as La_0.7_Ca_0.3_MnO_3_ and magnetite, Fe_3_O_4_, share similar physics as both have a spin-polarised conducting state near ambient temperature due to double exchange between ferromagnetically aligned Mn^3+^ and Mn^4+^ or Fe^2+^ and Fe^3+^ spins, respectively. Both undergo charge ordering on cooling, which for magnetite is accompanied by a lattice distortion below the much-studied Verwey transition at 125 K^1^. This was recently found to result from a complex ordering of Fe^2+^/Fe^3+^ charge states, Fe^2+^ orbitals, and three-Fe trimeron groups^2^. However, long range phase segregation is observed below charge ordering transitions in many manganites^3–5^ and related perovskites but has not been found in magnetite or related ferrite spinels.

Here, following recent studies of spin and charge ordering in non-spinel *M*Fe_*n*–1_O_*n*+1_ (*n* ≥ 4) ferrites with *M* = Fe^6,7^, Mn^8,9^ and Ca^10,11^, we investigate *n* = 4 CaFe_3_O_5_ and discover long range electronic phase separation between a magnetite-like charge ordered phase and a charge averaged phase.

## Results

### Preparation and characterisation of CaFe_3_O_5_

**Fig. 1:**
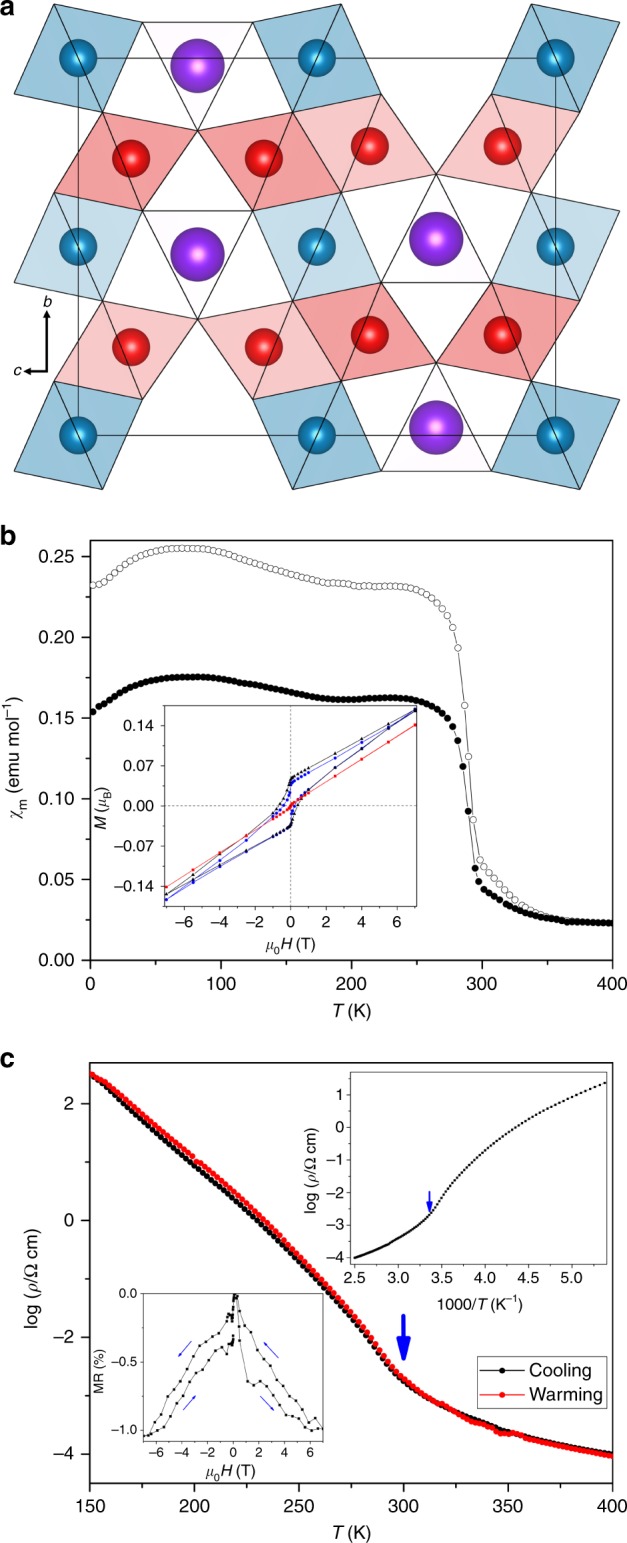
Crystal structure and magnetisation studies of CaFe_3_O_5_. **a** Polyhedral projection of the CaFe_3_O_5_ structure showing FeO_6_ octahedra in red/blue for symmetry inequivalent Fe1/Fe2 sites, and Ca within trigonal prismatic tunnels in purple. **b** ZFC (closed symbols) and FC (open symbols) magnetic susceptibilities for CaFe_3_O_5_, with insert showing magnetisation-field measurements at 2, 230 and 300 K (blue, black and red points respectively). **c** Log_10_ plot of the electrical resistivity of a sintered pellet of CaFe_3_O_5_ against temperature on cooling and warming, with the discontinuity at 302 K marked. Inset in the top right shows the plot against reciprocal temperature with a change in activation energy from 0.61 eV below *T*_M_ to 0.26 eV above the transition. Inset in lower left displays the magnetoresistance MR at 200 K

CaFe_3_O_5_ was synthesised and characterised as described in Methods. The orthorhombic crystal structure consists of FeO_6_ octahedra sharing edges to form infinite chains parallel to the *a*-axis and ribbons of three octahedra parallel to the *bc*-plane as shown in Fig. 1a. Magnetisation measurements (Fig. 1b) reveal a sharp magnetic ordering transition at *T*_M_ = 302 K and a small net magnetisation of 0.05 *µ*_B_ per CaFe_3_O_5_ unit with a moderate coercivity of 0.3 T at 2 K. Ceramic pellets of CaFe_3_O_5_ are semiconducting with a small magnetoresistance of −1% at 200 K (Fig. 1c), likely reflecting the small net magnetisation of the sample.

### Evidence for electronic phase separation in CaFe_3_O_5_

**Fig. 2:**
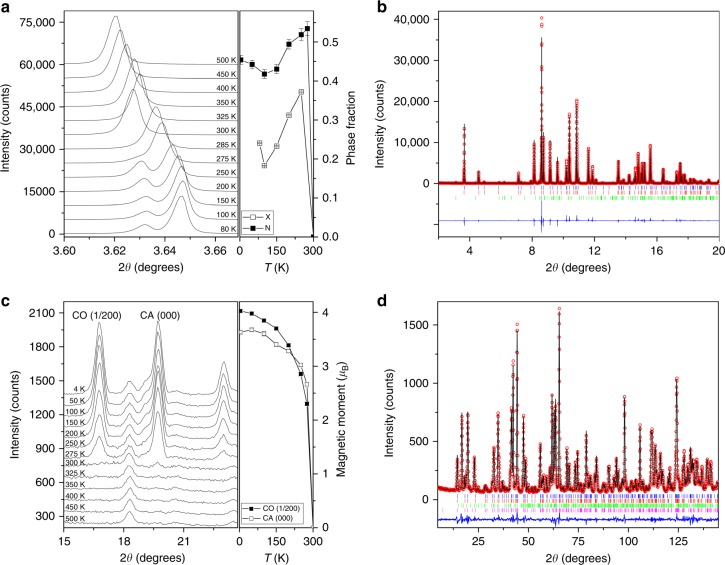
Electronic phase separation of CaFe_3_O_5_. **a** Evolution of the X-ray (002) peak which shifts to lower 2*θ* when cooled to 300 K, just below the *T*_M_ = 302 K transition, with separation into two components at lower temperatures. The phase fractions of the charge averaged (CA) phase, obtained from synchrotron (X) and neutron (N) diffraction data are shown in the right-hand panel. Error bars are the estimated standard deviations calculated during profile fitting. **b** Rietveld fit to synchrotron powder diffraction profiles for CaFe_3_O_5_ at 80 K (R-factors *R*_p_ = 9.35 %, *R*_wp_ = 11.0 %), with blue and red tick marks indicating the two low temperature phases and green marks 2.3% of Ca_2_Fe_2_O_5_ impurity. **c** The appearance of the magnetic reflections with propagation vectors of (000) for the CO phase and (½00) for the charge averaged (CA) phase below 302 K and the temperature evolution of their magnetic moments. **d** The Rietveld fit to neutron powder diffraction profiles for CaFe_3_O_5_ at 4 K (R-factors *R*_p_ = 6.14 %, *R*_wp_ = 7.28 %), with structural phases indicated with blue and red tick marks. The green and pink tick marks represent the magnetic phases with propagation vectors of (½00) and (000)

**Fig. 3:**
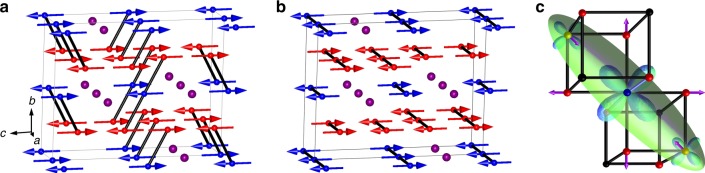
Magnetic structures for the two phases of CaFe_3_O_5_. Fe1/Fe2 spins are shown in red/blue and Ca ions in purple. **a** Magnetic structure of the charge ordered (CO) phase CaFe_3_O_5_ with propagation vector (½ 0 0), with lines showing ferromagnetic order within trimerons. **b** Magnetic structure of the charge averaged (CA) phase, with (0 0 0) propagation and lines showing the ferromagnetic chain parallel to the *x* axis. **c** A trimeron unit as found in the CO structure (**a**) with bonding electron density represented as an ellipsoid. The size of the t_2g_ orbitals approximates the atomic populations. The atomic displacement arrows indicate the elongation of the four Fe–O bonds perpendicular to the Jahn–Teller contracted axis and the shortening of the cation–cation distances due to weak Fe–Fe bonding interactions

A single high temperature (HT) crystalline phase of CaFe_3_O_5_ is observed above *T*_M_ = 302 K, but both powder synchrotron X-ray (Fig. 2a, b) and neutron diffraction data (Fig. 2c, d) reveal long range phase separation as diffraction peaks broaden or split into two components below the magnetic ordering transition as shown in Fig. 2a, c. The shift of the X-ray (002) peak to lower 2*θ* at 300 K evidences a small bulk lattice distortion due to spin ordering before separation into two phases at lower temperatures. Both low temperature phases have the same *Cmcm* space group symmetry as the HT parent. Crystallographic results are shown in Supplementary Tables 1–5. Magnetic neutron diffraction peaks observed below *T*_M_ (Supplementary Fig. 1) show that one phase has magnetic propagation vector (½ 0 0) while the other has (0 0 0) (Fig. 2c), and a good fit to the data (Fig. 2d) was obtained using the magnetic structure models shown in Fig. 3. No additional broadening of magnetic peaks was observed showing that both magnetic orders have correlation lengths of at least ~200 nm. Both phases have collinear antiferromagnetic orders with moments parallel to the *c*-axis, but an important difference is that the (½ 0 0) phase has ferromagnetic (FM) alignment of spins within the three-atom ribbons parallel to the *bc*-plane, while the (0 0 0) has FM spin chains parallel to the *a*-axis. Although both orders are antiferromagnetic, the observation of a small net magnetisation below *T*_M_ suggests that one or both of the spin structures are canted. The fractions of the two phases are found to be different in the synchrotron and neutron diffraction experiments (Fig. 2a), consistent with a strain-driven phase separation being dependent on the thermal history of the sample.

**Fig. 4:**
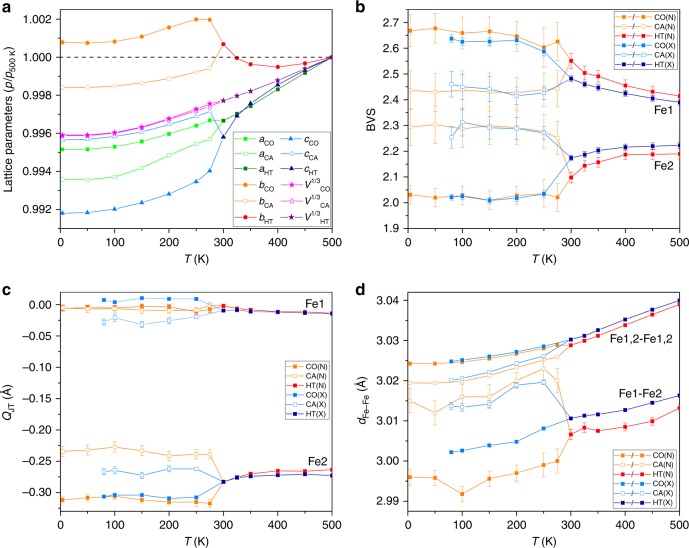
Crystal structure parameters of CaFe_3_O_5_. **a** Changes in the lattice parameters relative to 500 K values obtained from PND (*a*_500 K_ = 3.03896(1), *b*_500 K_ = 10.01355(5) and *c*_500 K_ = 12.67039(7) Å). Temperature dependence of the **b** BVS and **c** the amplitude of Jahn–Teller distortion (*Q*_JT_) of the FeO_6_ octahedra for the two sites in the high temperature (HT), charge ordered (CO) and charge averaged (CA) phases, obtained from neutron (N) and synchrotron X-ray (X) diffraction data. **d** The evolution of the Fe1-Fe2 bonding distance with temperature, with the lattice parameter (Fe1,2-Fe1,2) as reference. Error bars are the estimated standard deviations calculated during profile fitting

Thermal evolution of lattice parameters from neutron and X-ray fits are, respectively, shown in Fig. 4a and Supplementary Fig. 2, and variations of internal structural quantities from both studies are shown in Fig. 4b–d to emphasise the reproducibility of discovered structural differences between the two phases. Two inequivalent iron sites Fe1 and Fe2 are present in a 2:1 ratio in CaFe_3_O_5_, and in the HT phase they have respective Bond Valence Sums (BVSs) of 2.43 and 2.22, equivalent to formal charges of +2.75 and +2.50 when renormalized to the average of +2.67. Differing charge redistributions occur below the 302 K magnetic and phase segregation transition (Fig. 4b). In the phase with magnetic propagation vector (½ 0 0), the Fe1 and Fe2 BVSs diverge to very different values of 2.67(6) and 2.03(4) at 4 K, consistent with charge order (CO) of Fe^3+^ and Fe^2+^ respectively. The coexisting phase with (0 0 0) magnetic order shows the opposite behaviour as the Fe1 and Fe2 BVSs converge to similar values of 2.44(8) and 2.29(7) at 4 K showing that the electronic states at the two sites are not significantly different, hence this phase is charge averaged (CA).

Degenerate 3d^6^ Fe^2+^ states are subject to Jahn–Teller distortion which leads to tetragonal octahedral compression in magnetite^2^, corresponding to negative values of the *Q*_JT_ distortion parameter. The Fe2 site in CaFe_3_O_5_ has a large negative *Q*_JT_ value due to intrinsic distortions within the HT structure (Fig. 4c), but below *T*_M_ = 302 K *Q*_JT_ becomes more negative for the CO phase, consistent with localisation of Fe^2+^ states, while the magnitude of *Q*_JT_ decreases in keeping with the observed increase in Fe2 site valence for the CA phase. Jahn–Teller distortion at the Fe2 site in the CO phase leads to order of the t_2g_ orbital with minority spin electron density directed towards the two neighbouring Fe1 sites. Hence this localised orbital has the correct orientation to form a trimeron, a linear unit of three Fe ions formed by delocalisation of the minority spin electron in the ordered orbital of the central Fe^2+^ ion^2^, as shown in Fig. 3c. Trimeron formation requires FM alignment of the three magnetic moments, Fe^3+^–Fe^2+^–Fe^3+^ CO, orbital order at the central Fe^2+^ in the plane of the three atoms, and shortening of the Fe–Fe distances within the trimeron^2^. All these conditions are observed in the Fe1–Fe2–Fe1 ribbons in the CO phase with shortening of Fe1–Fe2 distances below *T*_M_ observed in Fig. 4d, but none are fulfilled in the CA structure, so the observed spin orders and structural distortions in CaFe_3_O_5_ demonstrate that trimeron order is observed only in the CO phase. However, a slight shortening of Fe–Fe distances in the FM chains parallel to the *a*-axis is observed in the CA phase (Fig. 4d), consistent with a weak bonding effect from extended band states of the minority spin electrons. Fe1–Fe1 and Fe2–Fe2 distances are constrained to be equal (to *a*/2) and this is the likely driver for the observed averaging of charge states between the two sites in the CA phase so that their minority spin populations become more equal.

The electronic orders in the two phases of CaFe_3_O_5_ do not change the structural symmetry, but they lead to different cell distortions (Fig. 4a) as trimeron formation shortens the *c*-axis parameter in the CO phase, with *a* and *b* expanding to compensate, while the *a*-axis shortening in the CA phase leads to expansion of *c*. Although the overall cell volumes for the two components remain identical within error at low temperatures, the differing spontaneous strains from these lattice distortions are the likely driver for the long range separation as proposed for perovskite manganites^12–14^. Short range phase and strain fluctuations in the HT phase are evidenced indirectly below 350 K through divergence of zero field- and field- cooled susceptibilities in Fig. 1b, and changes in the slopes of *b* and *c* lattice parameters in Fig. 4a.

Charge ordering in one of the two low temperature phases is clearly a key driver for the long range electronic phase separation in CaFe_3_O_5_ and perovskite type oxides^15^ although in CaFe_3_O_5_ this leads to the formation of orbital molecules^16^, more complex electronic objects than simple localised-charge ions. However, phase separation in CaFe_3_O_5_ occurs without a change of lattice symmetry in either component, demonstrating that strain variations within a given lattice are sufficient to drive long range segregation. Band structure calculations (shown in Supplementary Fig. 4 and Supplementary Table 6) confirm that these distortions are sufficient to stabilise CO in one structure but not the other for a realistic value of the Hubbard U-parameter. Phase separation in manganites usually results from an incomplete phase transition where a metallic HT FM phase is partially transformed to a charge ordered insulator, and the high and low temperature phases coexist to base temperature. A notable example is Nd_0.5_Sr_0.5_MnO_3_ which on cooling was observed to order as a single FM phase at 250 K, then to phase separate into a mixture of FM and A-type antiferromagnetic (AFM-A) phases below 220 K, and finally to undergo a further phase separation into a mixture of FM, AFM-A and charge ordered AFM-CE phases below 150 K, with all three phases extant down to the lowest measured temperature of 15 K^17^. CaFe_3_O_5_ undergoes a genuine electronic phase separation in the sense that both of the two low temperature CA and CO phases are electronically and magnetically distinct from the HT paramagnetic state, and so is analogous to e.g. the separation of a fluid into a liquid and gas below a critical transition. Both low temperature CaFe_3_O_5_ phases are magnetically ordered and it is remarkable that they appear to share a common magnetic ordering temperature as shown in Fig. 2c although the spin-spin exchange interactions within the two magnetic structures will not be identical.

Another important difference is that phase separation in manganites is associated with substantial intrinsic disorder due to cation mixing, and a recent study has demonstrated that chemical ordering of cations in (La_1−*y*_Pr_*y*_)_1−x_Ca_*x*_MnO_3_ suppresses long range phase separation^18^. CaFe_3_O_5_ is in principle a stoichiometric material although small strain variations due to the 4% substitution of Fe for Ca observed in our polycrystalline sample may tip the local balance between the energies of the two ground states leading to phase coexistence. The resulting disorder in magnetic interactions could also be important in stabilising the phase coexistence in a Griffiths-type model^19^. All Mn sites are electronically equivalent in the HT aristotype manganite perovskite structure but CaFe_3_O_5_ has a further electronic degree of freedom as charge can be redistributed between structurally inequivalent Fe1 and Fe2 sites, leading to the observed extremes of charge ordering (CO) in one phase and charge averaging (CA) in the other. This charge redistribution mechanism is akin to electronic separation models originally proposed for manganites^20,21^, although couplings of both the charge redistribution degree of freedom and trimeron formation associated with charge and orbital ordering to the lattice appear to be important factors that drive phase segregation in CaFe_3_O_5_.

## Discussion

In conclusion, the present study demonstrates that phase separation of a single HT paramagnetic state into two distinct low temperature phases with different long range spin and electronic orders occurs in CaFe_3_O_5_ when cooled below 302 K. Although both phases are antiferromagnetic overall, the formation of different FM units within them is coupled to the electronic orders; one phase has full Fe^2+^/Fe^3+^ CO associated with trimeron formation, but CA stabilises FM chains in the other phase. Weak Fe–Fe bonding driven by the FM orders introduces different lattice strains into the two phases although no change of structural symmetry occurs, and strain variations within the polycrystalline sample tip the local balance between the energies of the two phases leading to phase coexistence. CaFe_3_O_5_ thus links the trimeron-ordering of magnetite to the microstructural physics of perovskite manganites. CaFe_3_O_5_ also demonstrates new possibilities for more complex ‘electronically soft matter’^22^ than in perovskites where orbital molecule formation and redistribution of charge between distinct cation sites offer additional degrees of freedom for generation and potential control of electronic phase-segregated nanostructures.

## Methods

### Sample preparation and characterisation

Polycrystalline CaFe_3_O_5_ was prepared from stoichiometric quantities of CaFe_2_O_4_, Fe_2_O_3_, and Fe powders pressed into pellets, sealed in evacuated quartz tubes, and heated at 1100 °C for 12 h. (The CaFe_2_O_4_ was synthesised at ambient pressure using the ceramic technique outlined by Wan et al^23^. where CaCO_3_ and Fe_2_O_3_ powders were ground together in 1:1 ratio, pressed into pellets, heated at 850 °C for 4 h, reground and repelleted, and finally reheated at 1100 °C for 12 h.) Thermogravimetric analysis heating the sample in air at 10 °C min^−1^ to 900 °C, as shown in Supplementary Fig. 3, gave a mass increase of 2.789%, in agreement with the calculated value of 2.781% for oxidation of CaFe_3_O_5_. Powder X-ray diffraction confirmed that CaFe_3_O_5_ adopts an orthorhombic Sr_2_Tl_2_O_5_ type structure with space group *Cmcm* as reported previously^10^.

### Magnetic and electrical measurements

Magnetic measurements were carried out with a Quantum Design MPMS XL SQUID magnetometer. Magnetic susceptibility was recorded in zero field cooled (ZFC) and field cooled (FC) conditions between 2 and 400 K with an applied magnetic field of 1000 Oe. Hysteresis loops were also measured at 2, 230 and 300 K. Electrical resistivity measurements were carried out with a Quantum Design PPMS, between 180 and 400 K. Magnetoresistance hysteresis loops were also measured at 200 K.

### Powder synchrotron X-ray and neutron diffraction studies

High resolution powder X-ray diffraction data were collected at the ID22 beamline of the ESRF with incident wavelength 0.3999 Å. The powder was packed into a glass capillary with an outer diameter of 0.7 mm and spun during data acquisition with temperatures from 80 to 500 K controlled using an Oxford Cryostream system. High resolution neutron diffraction data were collected at the D2B beamline of the ILL with incident wavelength 1.5940 Å. 5 g of powder samples were packed into a vanadium can and diffraction patterns were collected at temperatures from 4 to 500 K. Crystal and magnetic structures of CaFe_3_O_5_ were Rietveld-fitted using the FullProf Suite^24^. A small amount of Fe at the Ca site was found from both synchrotron X-ray [4.4(3)% Fe] and neutron [4.0(8)% Fe] refinements of cation site occupancies at 500 K. Crystal structure refinements of the two phases formed below 302 K in CaFe_3_O_5_ was possible with both the synchrotron X-ray and neutron powder diffraction data except at temperatures just below the transition (275 and 285 K). Magnetic irreducible representation analysis was carried out using BasIrReps. Bond valence sums for each iron site were calculated using a standard method with linear interpolation to estimate mixed charge states between Fe^2+^ and Fe^3+^^25,26^. The crystal structure projection in Fig. 1a was generated using VESTA^27^ and the magnetic structures in Fig. 3a, b were made using FullProf Studio.

### Electronic structure calculations

DFT+U electronic structure calculations were performed using CASTEP^28^ (v16 and 17) utilising planewaves (650 eV cutoff) and on-the-fly pseudopotentials, within the PBE approximation to exchange and correlation. Band structures (shown in Supplementary Fig. 4 and Supplementary Table 6) predict metallic charge averaged ground states for both the refined CO and CA structures at small values of the Hubbard *U* energy, and insulating charge ordered states for both phases at large *U*. However, values in the range 2.0 < *U* < 4.0 eV simultaneously predict the charge averaged state for the CA structure and the charge ordered state for the CO structure, and hence confirm that this range is realistic for Fe oxides.

### Data availability

Data that support the findings of this study have been deposited at 10.7488/ds/2378.

## Electronic supplementary material

Supplementary Information
