# Author Correction: Long range electronic phase separation in CaFe_3_O_5_

**DOI:** 10.1038/s41467-018-06047-x

**Published:** 2018-08-28

**Authors:** Ka. H. Hong, Angel M. Arevalo-Lopez, James Cumby, Clemens Ritter, J. Paul Attfield

**Affiliations:** 1 Centre for Science at Extreme Conditions and School of Chemistry, University of Edinburgh, Mayfield Road, Edinburgh, EH9 3JZ UK; 2 Univ. Lille, CNRS, Centrale Lille, ENSCL, Univ. Artois, UMR 8181 - UCCS - Unité de Catalyse et Chimie du Solide, Lille, F-59000 France; 3 Institut Laue-Langevin, 71 avenue des Martyrs, Grenoble, 38000 France

Correction to: *Nature Communications* 10.1038/s41467-018-05363-6; published online 30 July 2018

The original version of this Article contained an error in the third sentence of the legend of Fig. 2, which incorrectly read ‘The phase fractions of the charge ordered (CO) phase, obtained from synchrotron (X) and neutron (N) diffraction data are shown in the right-hand panel.’ The correct version states ‘charge averaged (CA)’ in place of ‘charge ordered (CO)’. This has been corrected in both the PDF and HTML versions of the Article.

