# Supplementary material for: Long range electronic phase separation in CaFe3O5
Source: Nat Commun. 2018 Jul 30;9:2975. doi: 10.1038/s41467-018-05363-6 (PMC6065443; doi:10.1038/s41467-018-05363-6)
Supplement: Supplementary file 1 — Supplementary Information [file 41467_2018_5363_MOESM1_ESM.pdf]

Long range electronic phase separation in $CaFe_3O_5$

Ka. H. Hong, Angel M. Arevalo-Lopez, James Cumby, Clemens Ritter and J. Paul. Attfield

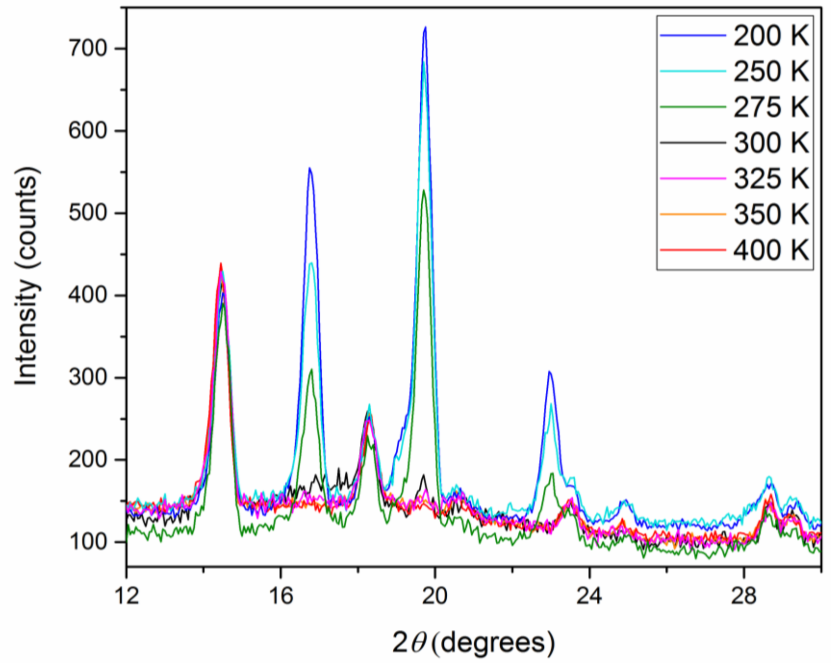

Supplementary Figure 1. Low angle region of powder neutron diffraction patterns of $CaFe_3O_5$. The thermal evolution of the magnetic scattering shows little change on cooling from 400 to 325 K, but some diffuse scatter is seen at 300 K, just below the onset of spin order at T_M = 302 K.

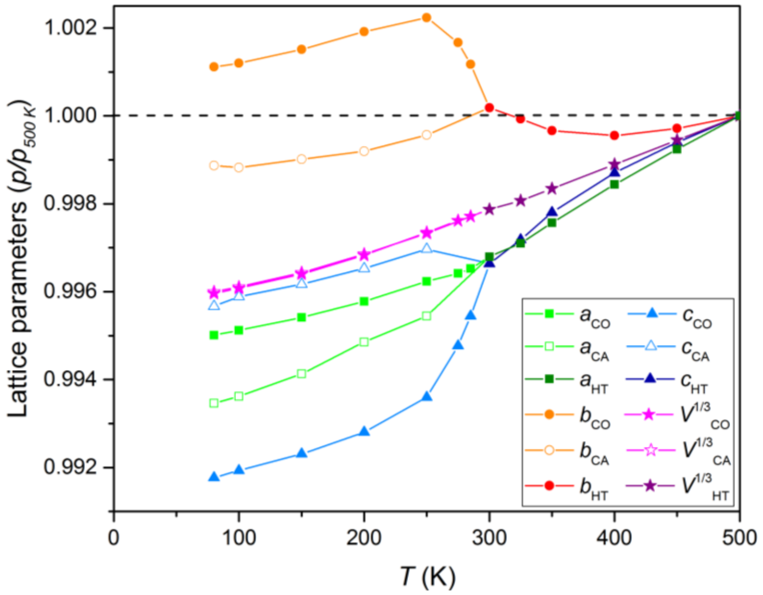

Supplementary Figure 2. Lattice parameters of $CaFe_3O_5$. Changes in the lattice parameters relative to 500 K values obtained from PSXRD data ($a_{500\ K}$ = 3.039972(2), $b_{500\ K}$ = 10.016679(8) and $c_{500\ K}$ = 12.676256(10)), showing similar variations to PND data in Fig. 4a.

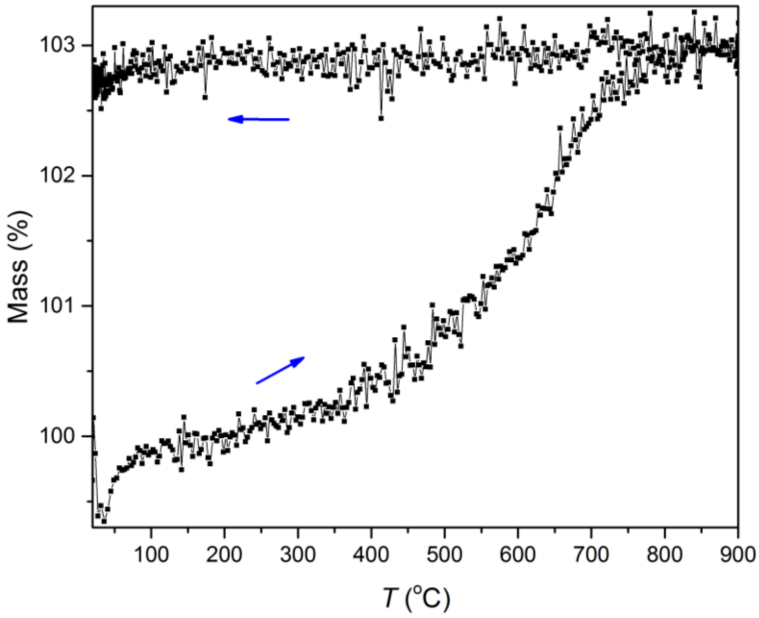

Supplementary Figure 3. Thermogravimetric analysis of $CaFe_3O_5$. The plot shows the change in percentage mass while heating the $CaFe_3O_5$ sample in air at 10 °C min^{-1} to 900 °C and then cooling at the same rate. Arrows indicate the direction of the measurement.

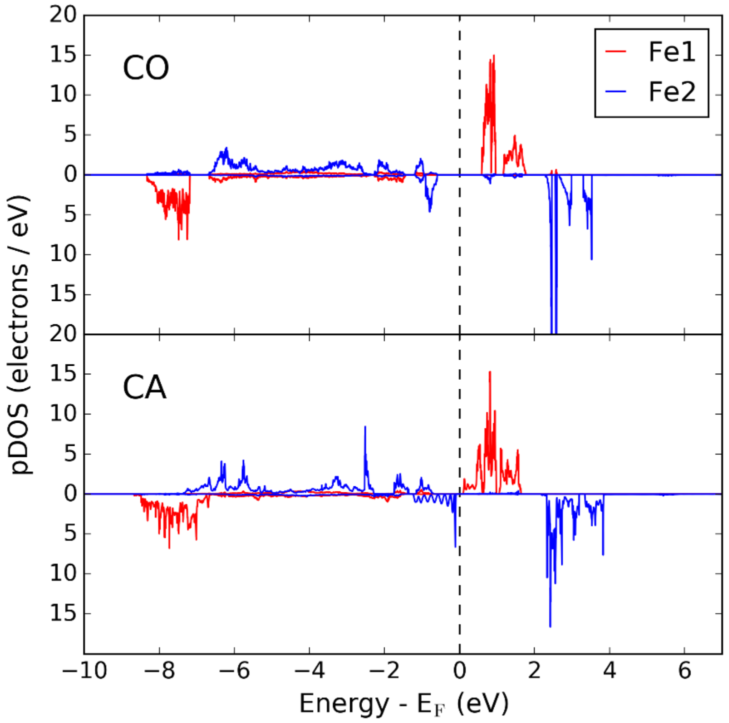

Supplementary Figure 4. Electronic density of states for $CaFe_3O_5$. Partial density of states (pDOS) for Fe1 and Fe2 d-orbitals (arbitrary spin direction chosen) for CO and CA phases, calculated with U = 3.4 eV. CO pDOS shows increased band mixing between Fe1 and Fe2 in the region around -8 eV, attributed to trimeron bonding.

Supplementary Table 1. Lattice parameters, atomic coordinates, and isotropic thermal displacements from neutron refinements in *Cmcm* space group of $CaFe_3O_5$ HT phase at 500 K (upper values) and CO and CA phases at 4 K (lower values). Estimated standard deviations in independent variables are shown in parentheses. *R* factors are R_{wp} = 7.70 and 7.28 %, and R_p = 7.32 and 6.14 % at 500 K and 4 K, respectively.

a /Å		*b* /Å		*c* /Å		Volume /Å^3	
HT		HT		HT		HT	
CO	CA	CO	CA	CO	CA	CO	CA
3.03896(1)		10.01354(5)		12.67038(7)		385.569(3)	
3.02425(3)	3.01944(3)	10.02139(10)	9.99766(11)	12.56654(10)	12.61533(15)	380.857(6)	380.823(7)

Atom	Site	*x*		*y*		*z*		Occupancy[a]		B_{iso} /Å^2	
		HT		HT		HT		HT		HT	
		CO	CA	CO	CA	CO	CA	CO	CA	CO	CA
Fe1	8*f*	0		0.26614(10)		0.11098(7)		1		1.18(2)	
		0	0	0.26496(20)	0.26688(27)	0.11038(13)	0.11125(17)	1	1	0.54(4)	0.56(6)
Fe2	4*a*	0		0		0		1		1.35(3)	
		0	0	0	0	0	0	1	1	0.71(5)	0.49(6)
Ca1/Fe	4*c*	0		0.52025(29)		¼		0.952(8)/0.040(8)		1.49(5)	
		0	0	0.52008(52)	0.51938(72)	¼	¼	0.952(8)/ 0.040(8)	0.952(8)/ 0.040(8)	0.60(9)	0.86(12)
O1	4*c*	0		0.19199(20)		¼		1		1.23(4)	
		0	0	0.19241(44)	0.19135(64)	¼	¼	1	1	0.51(8)	1.03(11)
O2	8*f*	0		0.64375(19)		0.44928(13)		1		1.62(3)	
		0	0	0.65048(32)	0.64088(56)	0.44839(23)	0.44849(30)	1	1	0.65(5)	1.17(8)
O3	8*f*	0		0.90437(19)		0.13509(11)		1		1.29(3)	
		0	0	0.90340(40)	0.90383(59)	0.13570(19)	0.13566(28)	1	1	0.62(6)	0.96(7)

[a] Variable occupancies were refined against 500 K neutron data and were fixed in lower temperature refinements. The refined Ca1/Fe occupancy from synchrotron X-ray diffraction data at 500 K is 0.956(3)/0.044(3).

Supplementary Table 2. Fe-O and Fe-Fe bond lengths (Å) obtained from powder neutron diffraction data, with mean values < >, shown for the HT phase of $CaFe_3O_5$ at 500 K (upper values) and the CO and CA phases at 4 K (lower values).

Bond	Distance		Bond	Distance	
	HT			HT	
	CO	CA		CO	CA
Ca1-O1 (x 2)	2.295(3)		<Fe1-O>	2.083(1)	
	2.296(5)	2.288(7)		2.055(1)	2.082(2)
Ca1-O3 (x 4)	2.403(2)		Fe2-O2 (x 4)	2.190(1)	
	2.296(5)	2.288(7)		2.231(3)	2.165(5)
<Ca1-O>	2.367(2)		Fe2-O3 (x 2)	1.961(2)	
	2.359(4)	2.354(6)		1.961(3)	1.962(5)
Fe1-O1	1.912(1)		<Fe2-O>	2.113(1)	
	1.899(3)	1.907(4)		2.141(1)	2.097(2)
Fe1-O2	2.239(2)		Fe1-Fe1, Fe2-Fe2	3.039(1)	
	2.205(4)	2.251(5)		3.024(1)	3.019(1)
Fe1-O2 (x 2)	2.096(2)		Fe2-Fe2(x 2)	3.213(2)	
	2.037(3)	2.106(5)		3.173(3)	3.205(4)
Fe1-O3 (x 2)	2.078(2)		Fe1-Fe2(x 4)	3.126(1)	
	2.077(4)	2.061(5)		3.124(2)	3.111(3)

Supplementary Table 3. Irreducible representations (IrReps) and basis vectors (BV) for Fe1 and Fe2 spin order in the CO phase of $CaFe_3O_5$ at 4 K, with propagation vector (½ 0 0). The magnetically independent atoms are Fe1 at (½, 0.2650, 0.1104) and Fe2 at (0, 0, 0). The symmetry related positions are generated by the operators 1: (*x*, *y*, *z*), 2: (*x*, *y*, -*z*+½), 3: (*x*, -*y*, *z*+½) and 4: (*x*, -*y*, -*z*). The structure was solved using $\Gamma_2\psi_4$, $\Gamma_2\psi_5$ and $\Gamma_2\psi_6$ for Fe1 and $\Gamma_2\psi_4$, $\Gamma_4\psi_{11}$ and $\Gamma_4\psi_{12}$ for Fe2, with R_p and R_{wp} factors = 6.1 and 7.3 %, respectively.

IrReps	Γ_1			Γ_2			Γ_3			Γ_4		
BV	ψ_1	ψ_2	ψ_3	ψ_4	ψ_5	ψ_6	ψ_7	ψ_8	ψ_9	ψ_{10}	ψ_{11}	ψ_{12}
Atoms	m_x	m_y	m_z	m_x	m_y	m_z	m_x	m_y	m_z	m_x	m_y	m_z
Fe1_1	+	+	+	+	+	+	+	+	+	+	+	+
Fe1_2	+	-	-	+	-	-	-	+	+	-	+	+
Fe1_3	-	-	+	+	+	-	-	-	+	+	+	-
Fe1_4	-	+	-	+	-	+	+	-	+	-	+	-
Fe2_1	+			+				+	+		+	+
Fe2_2	-			+				-	+		+	-

Supplementary Table 4. Irreducible representations (IrReps) and basis vectors (BV) for Fe1 and Fe2 spin order in the CA phase of $CaFe_3O_5$ at 4 K, with propagation vector (0 0 0). The magnetically independent atoms are Fe1 at (0, 0.2669, 0.1113) and Fe2 at (0, 0, 0). The symmetry related positions are generated by the operators 1: (x, y, z), 2: ($-x$, $-y$, z+½), 3: ($-x$, y, $-z$+½) and 4: (x, $-y$, $-z$). The structure was solved using $\Gamma_1\psi_1$, $\Gamma_5\psi_7$ and $\Gamma_5\psi_8$ for both Fe1 and Fe2 sites, with R_p and R_{wp} factors = 6.1 and 7.3 %, respectively.

IrReps	Γ_1	Γ_2		Γ_3		Γ_4	Γ_5		Γ_6	Γ_7	Γ_8	
BV	ψ_1	ψ_2	ψ_3	ψ_4	ψ_5	ψ_6	ψ_7	ψ_8	ψ_9	ψ_{10}	ψ_{11}	ψ_{12}
Atoms	m_x	m_y	m_z	m_y	m_z	m_x	m_y	m_z	m_x	m_x	m_y	m_z
Fe1_1	+	+	+	+	+	+	+	+	+	+	+	+
Fe1_2	-	-	+	-	+	-	+	-	+	+	+	-
Fe1_3	-	+	-	-	+	+	+	-	-	+	-	+
Fe1_4	+	-	-	+	+	-	+	+	-	+	-	-
Fe2_1	+			+	+		+	+		+		
Fe2_2	-			-	+		+	-		+		

Supplementary Table 5. The refined Fe moments in the CO and CA phases of $CaFe_3O_5$ between 4 and 300 K. Moments of the Fe1 and Fe2 sites were constrained to be equal.

Sites	Phases	4 K	50 K	100 K	150 K	200 K	250 K	275 K	300 K
Fe1/2	CO	4.03(3)	3.98(3)	3.85(3)	3.70(3)	3.39(3)	2.86(4)	2.30(4)	0
(μ_B)	CA	3.63(4)	3.68(4)	3.60(4)	3.40(4)	3.28(3)	3.02(3)	2.66(3)	0

Supplementary Table 6. Bader charge and volume, Mulliken charge and spin, and total energies of the CO and CA phases of $CaFe_3O_5$ from band structure calculations with U = 3.4 eV. Differences between Bader and Mulliken quantitities are in keeping with the greater charge separation between Fe1 and Fe2 sites in the CO phase compared to the CA found by BVS calculations as shown in Fig. 4b although magnitudes are smaller than expected from the formal valences.[1]

	Charge Ordered (CO)		Charge averaged (CA)	
	Fe1	Fe2	Fe1	Fe2
Bader Charge	1.85	1.51	1.83	1.55
Bader Volume ([bohr radius]^3)	50	59	52	56
Mulliken Charge	1.04	0.86	1.01	0.91
Mulliken Spin (μ_B)	4.08	3.72	4.07	3.65
Total energy (eV)	-46328.588		-46327.893	
Free energy (E – TS) (eV)	-46328.858		-46328.048	

Supplementary References

1. Pickett, W. E., Quan, Y. & and Pardo, V. Charge states of ions, and mechanisms of charge ordering transitions. *J. Phys.: Condens. Matter* **26**, 274203 (2014).
